# The Influence of the sEMG Amplitude Estimation Technique on the EMG–Force Relationship

**DOI:** 10.3390/s22113972

**Published:** 2022-05-24

**Authors:** Simone Ranaldi, Giovanni Corvini, Cristiano De Marchis, Silvia Conforto

**Affiliations:** 1 Department of Industrial, Electronics and Mechanical Engineering, Roma Tre University, 00154 Roma, Italy; simone.ranaldi@uniroma3.it (S.R.); giovanni.corvini@uniroma3.it (G.C.); 2 Department of Engineering, University of Messina, 98158 Messina, Italy; cristiano.demarchis@unime.it

**Keywords:** sEMG processing, force estimation, isometric contractions

## Abstract

The estimation of the sEMG–force relationship is an open problem in the scientific literature; current methods show different limitations and can achieve good performance only on limited scenarios, failing to identify a general solution to the optimization of this kind of analysis. In this work, this relationship has been estimated on two different datasets related to isometric force-tracking experiments by calculating the sEMG amplitude using different fixed-time constant moving-window filters, as well as an adaptive time-varying algorithm. Results show how the adaptive methods might be the most appropriate choice for the estimation of the correlation between the sEMG signal and the force time course. Moreover, the comparison between adaptive and standard filters highlights how the time constants exploited in the estimation strategy is not the only influence factor on this kind of analysis; a time-varying approach is able to constantly capture more information with respect to fixed stationary approaches with comparable window lengths.

## 1. Introduction

Surface ElecroMyoGraphy (sEMG) has been widely used as a means for measuring muscle activity during force generation, typically relating the amplitude of the sEMG signal to the amount of force exerted during a particular movement [1,2,3,4,5]. In most adopted models of the relationship between muscle activity and force, the capability of the contraction in generating a torque at a joint is dependent on the length of the muscle itself and its rate of change [6,7]; for this reason, the sEMG–force relationship is typically studied only in isometric conditions [8]. Even in this limited scenario, considering the different influencing factors on sEMG amplitude, as well as the numerosity of the muscles acting on each joint, the relationship between sEMG amplitude and generated force is approximately linear only below a certain level of force [9]. These models apply to static or quasi-static contractions, during which the time relationship is not considered, but when there is a need to model time-varying behaviors, and electromechanical delay (i.e., the delay between EMG and force onsets) must be taken into account. Considering the multitude of sources of noise in the investigation of the sEMG–force relationship, it is crucial to have a robust and thorough processing schema for information extraction from the biological signals, to be able to isolate fine characteristics that can be assigned to the correct influence factor, considering the particular experimental scenario [10]. Although it can be argued that this is a very general problem in sEMG-related experiments, the scientific literature still fails to have a simple and powerful model relating single-muscle sEMG recordings and force output during different conditions, suggesting that a more detailed characterization of the models and methods at the core of these analyses is needed.

Estimating the amplitude of the sEMG signal is a critical step in most of the well-established clinical and research analyses [1,11]; this computation is however often carried out by means of highly subjective methods, and the effect of different estimation strategies on the target parameters is unknown and uncharacterized. In the particular case of force estimation, since sEMG amplitude has been related to motor-unit synchronization [12], the envelope extraction phase is the main analytical tool to be applied on the signal. Moreover, synaptic noise is also present at the source of the signal (i.e., the neural drive) [13], so that optimal filtering is essential in managing different components of the noise.

sEMG envelope estimation is performed by low-pass filtering the rectified version of the raw signal, at cutoff frequencies that vary approximately in the range 2–20 Hz for general applications [14,15,16,17]; for isometric and quasi-static contractions, the optimal value has been typically considered to be in the very low-frequency portion of this range, considering that the sEMG amplitude can be hypothesized to have a frequency content below 5–10 Hz in these scenarios [18], with optimal cutoff frequencies around 2–3 Hz [5]. Filtering is achieved either by IIR filtering or by moving-window algorithm, the latter technique being more general in terms of its applicability also in online processing [19]; although the particular filtering strategy can reasonably be supposed to have small effects on outcomes, the different cutoff frequencies (or window lengths) strongly affect the smoothness and the responsiveness of the amplitude estimator, potentially affecting the correlations among estimated sEMG amplitude and any target signal, such as force variations.

In this work, different sEMG amplitude-estimation methods are tested on two different datasets to investigate whether choices in the processing schema and particularly in the filters used for sEMG envelope extraction yield results that are characterized by different correlation levels with the force signal, hence being more or less suitable to be used in sEMG–force relationship analyses.

## 2. Materials and Methods

### 2.1. Datasets

For this study, data coming from two different isometric force-tracking experiments have been used. In detail, the two experiments are related to isometric contractions of both upper limb (*triceps brachii lateral head*) and lower limb (*tibialis anterior*) muscles. Both tasks have been selected to have an experimental condition in which most of the force is generated by the recorded muscle alone, with minimal contribution from muscles belonging to different anatomical groups. Both experimental procedures have been approved by the local ethics committee of Roma Tre University.

#### 2.1.1. Experimental Protocol for the *Triceps* Experiment

**Figure 1:**
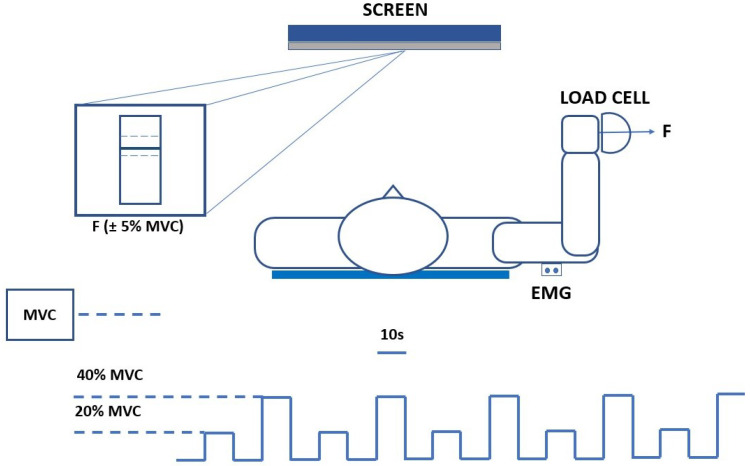
A visual representation of the experimental setup and the *triceps brachii* force-tracking protocol.

For these datasets, 16 healthy subjects (all males, 28 ± 2 years old, height 179 ± 10 cm, weight 82.4 ± 10.2 kg), righthanded were enrolled. Subjects were asked to track a force signal composed by the cyclical repetition of 10 s contractions at 20% and 40% of their maximum voluntary contraction (MVC), separated by a 5 s resting period at a very low force level (10% MVC). Visual feedback was realized by showing a colored line that the subjects had to keep within two limits corresponding to the desired value ±5% MVC (Figure 1).

During the whole task, sEMG signals are recorded with a wired StepPC system (DEMItalia) and synchronized with the force values recorded with a custom-made load cell (sensitivity: 10 mV/N, full-scale range: 2000 N). The visual stimulation signal is provided and recorded through a custom LabView panel that manages the whole data acquisition. Sampling frequency has been set to 1 kHz for both sEMG and force signals.

#### 2.1.2. Experimental Protocol for the *Tibialis* Experiment

Ten healthy subjects were enrolled for the *tibialis* experimental protocol (8 females and 2 males, 27 ± 3 years old, height 170 ± 8 cm, weight 64.3 ± 13.9 kg). Participants were asked to avoid any kind of fatiguing activity the day before the measurements.

**Figure 2:**
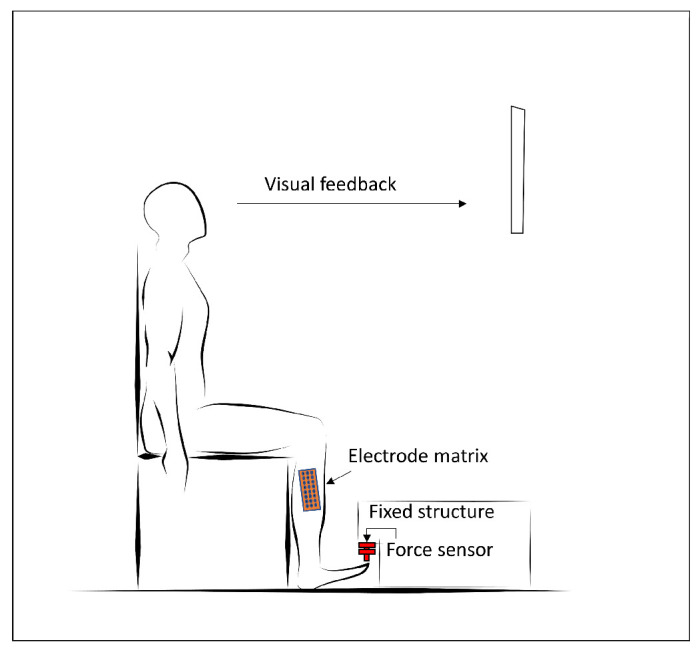
A visual representation of the experimental setup for the *tibialis anterior* force-tracking protocol.

A visual representation of the experimental protocol is given in Figure 2. Subjects were comfortably seated with the knee at a 90 degrees flexion angle. The dominant foot was placed under a fixed structure containing the force sensor. Heel was kept fixed to the ground during the whole experiment. The experiment consisted of a series of 5 contractions lasting 2 min at a 50% MVC force level. Appropriate resting periods between contractions were inserted between trials to avoid the presence of fatigue at the beginning of the successive trial. Visual feedback was provided to the subject by showing on a screen the time-varying force trace superimposed to the reference values.

Surface electromyography signals of the *tibialis anterior* muscle have been recorded through a high-density EMG (HD sEMG) sensor (SESSANTAQUATTRO, OTBioelettronica, Turin, Italy); from the HD sEMG data, a bipolar signal was selected to mimic in the best way SENIAM recommendations for sEMG recordings. Both sEMG and force signals were acquired at a sampling frequency of 2 kHz. Force signals were recorded with a compression load cell with a full-scale range of 220 N (FC2231-50L, TE Connectivity, Schaffhausen, Switzerland).

### 2.2. sEMG Processing

The processing strategy for the sEMG signal was standardized for both the experimental protocols. In more detail, as a first step the sEMG signal was pre-processed using standard denoising techniques (3rd-order Butterworth band-pass filter between 25 and 450 Hz and 3rd-order Butterworth notch filter at 50 Hz with a 1 Hz bandwidth), prior to envelope extraction.

The sEMG envelope has then been extracted via rectification and a moving RMS filter with different window lengths, namely 500, 200, 100, 80, 66 and 44 ms ($MW_{500}$, $MW_{200}$, $MW_{100}$, $MW_{80}$, $MW_{66}$ and $MW_{44}$). In addition, the adaptive envelope computed with the algorithm in [20] has been inserted in the analysis ($MW_{ADA}$), to test the effect of a time-dependent time constant in the estimation of the sEMG–force relationship.

The adaptive algorithm works via iteratively adapting a sample-by-sample window length for an RMS filter. The optimization target is the minimization in the RMS error in the estimation of the amplitude of the sEMG signal, and the convergence criterion is based on the evaluation of the estimation entropy.

The SNR for each event has been calculated via the inverse of the coefficient of variation of the sEMG amplitude during the contraction phase. Given its optimality in terms of information extraction, the results from the adaptive algorithm have been used for this estimation

$$SNR_{dB}=10\log_{10}\frac{\mu_{ADA}}{\sigma_{ADA}}$$

The signal and noise power has been calculated by considering the particular problem to be solved; in this sense, $\mu_{ADA}$ is the mean value of the envelope during the contraction, and $\sigma_{ADA}$ is its variability.

This estimation has been carried out by analyzing a 1 s window starting 4 s after the contraction onset, for each event of each subject. Average values across all events and all subjects were taken as a general estimation of the SNR in the different tested conditions.

### 2.3. sEMG–Force Relationship Measures

The quality of the estimated relationship has been tested by analyzing the contraction and release phases separately.

The phases have been defined differently for the two datasets:

- *Triceps*. For this dataset, the events have been defined starting from the signal related to the visual stimuli that has been given to the subject during the experiment.
- *Tibialis anterior*. For this dataset, events have been defined directly from the force signal, defining a threshold based on the noise level.

In both datasets, the segments related to onsets and offsets have been defined ranging from 0.5 s before the event, with a 2.5 s total duration.

Considering that most of the models for sEMG–force relationship are linear in nature [4], for both phases the correlation coefficient between the force signal and the sEMG envelope has been calculated as a quality parameter. The correlation coefficient has been calculated at its peak value from the cross-correlation function, in order not to take into account the delays induced by the filtering and the physiological electromechanical delay, which might insert trends into the results that are not indicative of the quality of the sEMG–force relationship.

In addition to the correlation measures, the root mean square error (RMSE) between the normalized time course of the force and the sEMG has been computed [21] as the RMS value of the difference between the two signals. Before calculating this parameter, the two segments were aligned to the delay corresponding to the maximum correlation, to compensate for physiological or instrumental time delays between the two quantities, such as the electromechanical delay [5].

The statistical significance of the differences between algorithms in the performance indicators has been tested by means of a one-way ANOVA test, with the algorithm as a factor. ANOVA assumptions were tested by checking the normality of the residuals of the model with a Shapiro–Wilk test. Three different tests were carried out separately—two for both force levels of the *triceps* dataset and one for the *tibialis* signals. Post hoc analysis was carried out with a Tukey test.

## 3. Results

### 3.1. Signal to Noise Ratio

**Figure 3:**
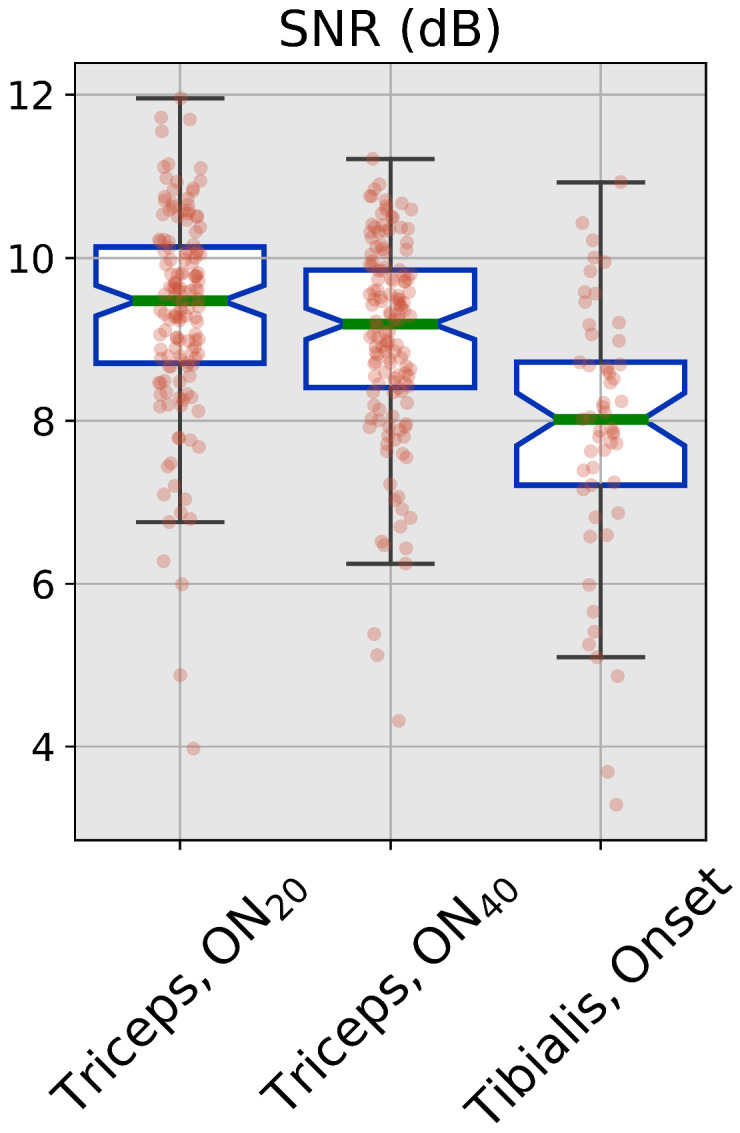
SNR values for the three tested conditions. Green line represents the median, blue lines are the inter-quartile range. Red dots refer to the single values.

The SNR values for the three conditions are shown in Figure 3. SNR levels for data coming from the *tibialis* dataset are significantly lower than all the values recorded for the *triceps* experiment (p<0.05, Wilcoxon Rank-Sum test with Bonferroni correction for multiple comparisons). No statistical difference has been recorded for the two force levels of the *triceps* experiment.

### 3.2. Time-Varying Filter Time Constant

**Figure 4:**
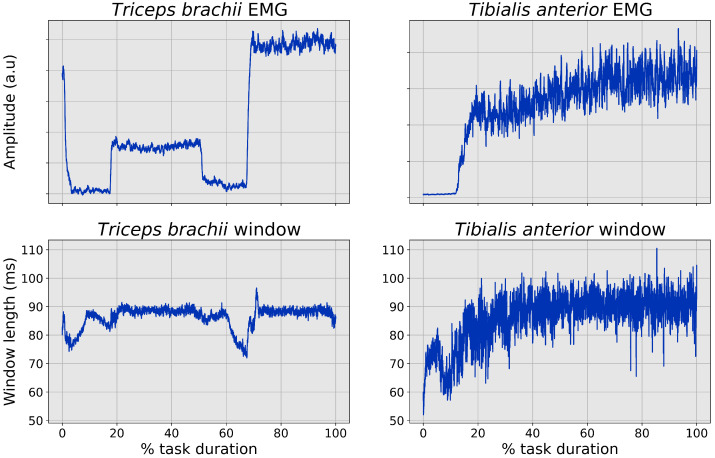
Average sEMG envelope and time-varying time constant behavior for the two datasets.

The average behavior, over all repetitions and all subjects, of the time-varying filter window length is shown in Figure 4 for both datasets. For the *triceps* dataset, a complete cycle is shown (both 20% and 40% force levels). For the *tibialis* data, before calculating the average behavior, all the trials have been interpolated over a fixed number of points (30,000 samples), considering the variable duration of the experimental trial; this step has been performed only for visualization purposes, not for the calculation of the actual correlation parameters.

The experimental protocols described in the Methods section resulted in an average of eight events for each *triceps* subject and five for the *tibialis* participants.

In both datasets, the time-varying filter time constant shows pronounced minima in correspondence to the onset of the contractions. Moreover, in both scenarios, the filter window stabilizes at a value of 90 ms during static or quasi-static contraction phases. Dataset-related differences that can be identified are:

- For the *tibialis* dataset, the minimum window length is around 60 ms, different to the 70 ms value that is recorded on the *triceps* signal;
- The variability of the *tibialis* window length is higher, with a noise level that is close to the actual range of the *triceps* dataset;
- For the *triceps* dataset, in which also a contraction offset phase is present, the same local minima for the window length can be found;
- In the *triceps* dataset, the local minima are more pronounced for higher-level contractions, both for onset and offset phases.

### 3.3. Correlation Metrics

For all the tested conditions, there is a significant worsening of the performance of the fixed-window algorithms for shorter time windows. In general, results on the *tibialis* dataset show lower correlation values and higher variability, while still maintaining the same trend. The adaptive algorithm performs with comparable correlation values (p>0.05) to the best-performing algorithms ($MW_{500}$ and $MW_{200}$) on the *tibialis* dataset and on the high force level of the *triceps* data; for lower force value, $MW_{ADA}$ performs significantly worse than $MW_{500}$ (p=0.03).

**Figure 5:**
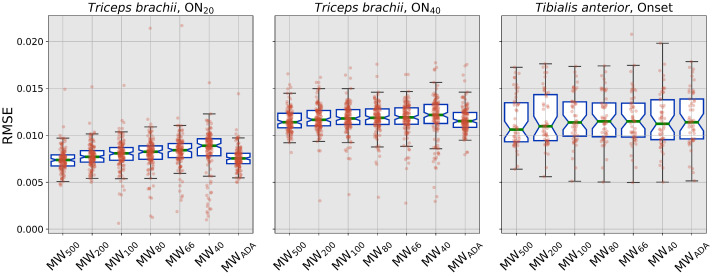
RMSE values. Colors have the same meaning as in Figure 3.

Similar results can be found on the RMSE values coming from the same analysis, as reported in Figure 5. For this parameter, the same significant differences can be identified in the *triceps* data, while no differences are present on the *tibialis* results.

## 4. Discussion

In this study, two simple force-tracking experiments have been exploited to test the effect of the processing choices (i.e., the amplitude-estimation strategy) on the evaluation of the sEMG–force relationship. Data from submaximal isometric contractions have been processed and tested in terms of the correlation values between the estimated amplitude envelope of the sEMG signal and the force curve during the onset phases and of the RMSE between the two signals. The set of classical fixed-time approaches presented here represents a subset of the methods that have been applied in the literature; the results coming from this analysis can be reasonably extended to other methods that need to be selected with some optimality criteria before being applied to sEMG–force estimation analyses.

In this work, we designed two experiments to have a condition in which all the force is generated by a single muscle; this condition is far from being often encountered in typical experimental scenarios. However, the multi-muscle force-generation condition requires some mathematical models to be built on top of the envelope-estimation procedure, so that these results presented here can be generalized to these wider conditions.

The tested methods are divided into standard, fixed-time constant moving-window procedures, and the adaptive procedure introduced in [20]. It has been shown that this latter algorithm can capture information not only in its main output (i.e., the sEMG envelope), but also in the time-dependent behavior of the estimated optimal time constant for the moving-window filter. In both the scenarios shown in Figure 4, it is evident how, for these slowly varying signals, this feature of the algorithm is also preserved. From a purely signal-feature point of view, the data from the two datasets are different in terms of rate of force change (i.e., the time to reach the target force from the baseline condition), sampling frequency and noise level; however, even with this inhomogeneous characteristic of the dataset, it is possible to identify a strongly repeatable minimum in the window length when the force level (and consequently the signal amplitude) abruptly changes.

In addition, the difference between the steady-state value for the time-varying window and the local minima at the onsets are different as a function of the force output that is generated by the contraction. When analyzing data only coming from the *triceps* dataset, this is true for both the onset and offset phases; data in the *tibialis* dataset are characterized by a higher force output (expressed in terms of fractions of MVC), thus justifying the shorter time windows that are recorded at the onset.

**Figure 6:**
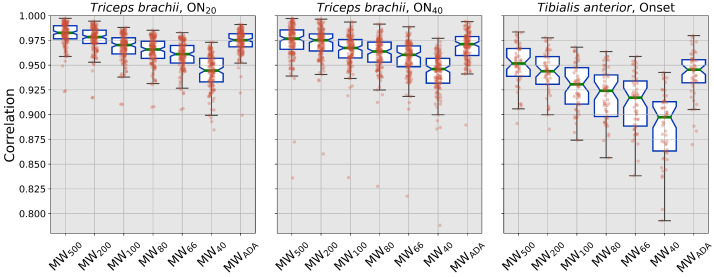
Correlation values. Colors have the same meaning as in Figure 3.

As an additional difference between the two experiments, it can be noted from Figure 3 that data from the *tibialis* dataset have a higher noise power with respect to *triceps*; the difference in SNR is clearly visible in the *plateau* phase of the contraction in Figure 6. Even in this high-noise scenario, the same trend for the correlation parameters can be identified, supporting the optimality hypothesis for the adaptive estimation of the sEMG envelope. RMS values do not show any difference across the two algorithms as a consequence of the high noise that is present close to the plateau region for the contraction. However, this high variability in the results is also present for the slower filter, which tends to filter out any high-frequency noise; considering this, it is reasonable to suppose that the absence of the same trend for RMSE values over the *tibialis* dataset is not to be ascribed to worse performance of the algorithms, but only to a more challenging condition in the dataset itself. It should be noted that this difference in SNR is not only to be ascribed to the recording and experimental settings, but it is indeed coming from the signal-dependent nature of the noise itself [22,23]; when the effects of the signal-dependent noise model are not negligible, the adoption of an optimal algorithm for the estimation of the sEMG amplitude has an even higher importance for ensuring the correctness of the results.

In all cases, the time dynamics identified by the adaptive algorithm is enclosed in the range 60–100 ms, which is completely included in the range that has been tested using the fixed-window methods (44–500 ms). However, in terms of correlation with the force, values coming from the fixed-window approach with time dynamics closer to $MW_{ADA}$ (i.e., $MW_{66}$, $MW_{80}$ and $MW_{100}$) are slightly or significantly lower than the ones relative to the adaptive approach. Although for the dataset coming from the *triceps brachii* contractions the values are still very high (>0.95 for all the methods except for $MW_{80}$), on a noisier and generally less standardized dataset, such as the one coming from the *tibialis* experiment, the differences are more evident, with the only methods that are close to the 0.95 correlation value being the two slowest fixed filters ($MW_{500}$ and $MW_{200}$) and the adaptive algorithm. All these trends in the results strongly suggest that, when analyzing the sEMG–force relationship, even if the task has slow dynamics, the time constant of the filters is not the only processing choice that influences the results.

As a general consideration, the slowly varying nature of the force-tracking experiments that have been analyzed here puts the slower filters in a clear advantage with respect to the shorter time windows; moreover, the nature of the quality parameter (i.e., the correlation) is intrinsically higher for time signals that come from very long time windows that are consequently very smooth. Even with this advantage, and even by estimating an envelope that is less smooth, $MW_{ADA}$ is comparable to those optimal solutions; as a consequence of this result, it is reasonable to suppose that the behavior of the adaptive algorithm is more consistent across different scenarios, in which the amplitude of the sEMG signal is varying with faster dynamics.

In this paper, the analysis has been focused on simple yet crucial quality parameters for the estimation of the sEMG–force relationship, namely the maximal correlation value and the RMSE, which has already been proven to be effective in characterizing the quality of force estimation from sEMG [24]. These parameters are focused on the characterization of the shape identification capabilities of the different methods, without quantifying the information about the timing error in the onset detection. Although it is possible to also quantify this feature of the sEMG relationship (possibly also exploiting the signal related to the time-varying window length from $MW_{ADA}$), such an analysis requires that several influence factors such as the electromechanical delay are taken into account, requiring complex and controlled experimental scenarios. Moreover, onset identification is a well-known problem in the scientific literature on sEMG signal processing, which already has optimal and widely accepted solutions. In this work, the focus has been put on identifying general results in a highly uncontrolled scenario using very simple experimental procedures and quality parameters, to identify general trends that can pave the way to an optimization of the sEMG processing choices for force-estimation applications.

Considering the fact that, for the *tibialis* dataset, the signals were extracted from an HD sEMG recording, the results presented here can be considered to be valid also in the case in which the sEMG–force relationship is exploited by this recording technology [3,21,25]. Estimating the amplitude of the different HD sEMG channel signals via an adaptive procedure can reasonably improve the outcomes of any processing algorithm that estimate force-related measures starting from the sEMG amplitude, yielding analogous performance differences with respect to the compact correlation measure that has been tested in this work using single-channel recordings.

An accurate estimation of the sEMG–force relationship can yield relevant parameters not only during the onset and offset phases. For example, it has been demonstrated that the force output is characterized by an increasing variability in the presence of neuromuscular fatigue [26,27,28], so that, with very high correlations between the force output and the sEMG signal, it can theoretically be possible to also identify this feature from the sEMG alone, giving rise to the definition of novel fatigue indicators. When neuromuscular fatigue is present, the sEMG signal has been shown to have increased amplitude and a spectrum that is more focused in the low-frequency region; although the presented results show that the performance of $MW_{ADA}$ are consistent across different force level, its mathematical assumptions and the presence of a pre-whitening filter in the algorithm ensure that the shift in the main frequency components have little or no effect in the final amplitude estimation.

In addition to improve fatigue detection, a repeatable, stable and reliable estimation of the sEMG–force relationship is also able to highlight more advanced and detailed information on force-generation mechanisms and force control in general, such as frequency and coherence behavior [28,29,30] or responses to visual stimuli in force-tracking procedures [31]. Both these scenarios are typically investigated in terms of very low-frequency oscillations (less than 1 Hz); the adoption of accurate and optimal amplitude-estimation algorithms might ensure that this information is captured in a stable manner even if no ad hoc very steep and narrow low-pass filters are adopted. Here, we focused on the analysis of consistent changes to the force level (i.e., the onsets); however, it is reasonable to suppose that the same advantage of the adaptive algorithm can also be recorded in the isotonic phase of the contraction, during which different mechanisms (e.g., fatigue itself) might result in small changes to the force level.

Although the results presented here are relative to a well-established research question (i.e., sEMG-based force estimation and tracking) and have strong physiological models underlying the experimental design [7], the considerations that have been made in this work are easily generalized to any application in which the key objective is to track dynamic changes to sEMG amplitude to estimate biomechanically relevant time-varying quantities. In all those cases, the adoption of an adaptive optimal procedure is reasonably the safest choice in being able to capture the wider portion of the relevant variability in the sEMG amplitude. 

## Data Availability

Data used in this study are available from the corresponding author under reasonable request.

## Abbreviations

The following abbreviations are used in this manuscript:

sEMG	Surface ElectroMyoGraphy
HD sEMG	High-Density Surface ElectroMyoGraphy
MW	Moving Window
MVC	Maximum Voluntary Contraction
RMS	Root Mean Square
RMSE	Root Mean Square Error
